# The Solidification/Stabilization of Wastewater (From a Landfill Leachate) in Specially Designed Binders Based on Coal Ash

**DOI:** 10.3390/ma14195610

**Published:** 2021-09-27

**Authors:** Carmen-Lidia Oproiu, Georgeta Voicu, Alina Bădănoiu, Adrian-Ionuţ Nicoară

**Affiliations:** Department of Science and Engineering of Oxide Materials and Nanomaterials, Faculty of Applied Chemistry and Materials Science, University POLITEHNICA of Bucharest, 1-7 Gheorghe Polizu Street, 011061 Bucharest, Romania; carmen_oproiu@yahoo.com (C.-L.O.); alina.badanoiu@upb.ro (A.B.); adrian.nicoara@upb.ro (A.-I.N.)

**Keywords:** solidification/stabilization, landfill leachate, coal ash, heavy metals

## Abstract

The aim of this study is to assess the possibility to solidify/stabilize a liquid waste from a municipal waste landfill using binders based on coal ash (fly ash and bottom ash) and specially designed cements for waste treatment (INERCEM). The leaching test proved that all cementitious systems are efficient for the solidification/stabilization of the studied wastes and can reduce the leaching potential of heavy metals present in both liquid waste and coal ash. Therefore, these wastes cease to be a source of environmental pollution. X-ray diffraction (XRD) and thermal complex analysis (DTA-TG) were used to assess the nature and amount of compounds formed in these cementitious systems during the hydration and hardening processes; ettringite, calcium silicate hydrates and CaCO_3_ were the main compounds formed in these systems assessed by these methods. The microstructure of hardened specimens was assessed by scanning electronic microscopy (SEM); the presence of hydrate phases, at the surface of cenospheres present in fly ash, proved the high pozzolanic reactivity of this phase.

## 1. Introduction

Romanian waste management legislation, in agreement with European legislation, establishes the framework for setting up a landfill, i.e., design, construction, operation, monitoring, closure and post-closure of the landfill, in terms of environmental protection and public health. It also details the management of the leachate collected from such deposits [1,2].

Landfill leachate is defined as the “liquid that has seeped through solid waste in a landfill and has extracted soluble dissolved or suspended materials in the process” [3]; in municipal solid waste landfills, this leachate is collected and removed from landfills for treatment and safe disposal [4].

Nowadays, most landfills process this liquid waste by a reverse osmosis process, in order to clean it before releasing it in a natural receptor. However, these reverse osmosis installations are sometime inefficient because the leachate has a very high concentration in organic/inorganic matter. In this case, the liquid from the reverse osmosis station (wastewater) is sent back to the landfill. In this way, not only is the issue is not solved, but the concentration of pollutants in the leachate continues to increase.

One possible alternative to safely dispose of this wastewater/leachate is to treat it by solidification/stabilization (S/S). S/S is an efficient, economically feasible and extensively used treatment for the management/disposal of a wide range of wastes [5]. This method consists of mixing the waste with binding agents such as lime, cement, gypsum, slag, fly ash or phosphate cements [5,6,7,8,9,10,11].

Fly ash and coal ash are by-products (wastes) that result in coal combustion in thermal power plants [12,13]. Depending on the coal type and source, the chemical composition of these ashes can vary, but the main components are silica, alumina, iron, calcium, alkalis and in some cases also heavy metals [12,13,14,15,16]. Fly ash, which is collected in the dust filters of the coal combustion installation, has physical and chemical characteristics that permits its valorization in the production of various inorganic binders [12,13,14,15,16,17,18,19]; on the opposite side, bottom ash is often disposed of in landfills, and due to its higher content in heavy metals (compared with fly ash) can represent a serious environmental problem [12,20].

The aim of this study was to find a feasible technological solution for the safe disposal of wastewater from the reverse osmosis treatment of a landfill leachate that cannot be released in natural receptors due to its high content of heavy metals. This wastewater was solidified/stabilized (S/S) in a cementitious matrix based on coal ash (considered also as hazardous waste due to its high content in heavy metals) and commercially available cements (INERCEM) specially designed to be used for waste/contaminated soil treatment.

## 2. Materials and Methods

The materials used in this study were as follows:

- Two types of commercially available cements, INERCEM A and INERCEM E, products specially designed to be used for waste/contaminated soils treatment (Holcim Romania SA, Campulung, Romania).
- Fly ash and bottom ash from a Romanian thermal power plant that uses solid fuel, mainly coal, using a controlled combustion at 800 °C for electrical energy production.
- Leachate formed in a solid municipal waste landfill that was treated by a reverse osmosis process. The resulted wastewater was used as the liquid component in the studied materials.
- Natural quartz sand (Societe Nouvelle du Litoral, Leucate, France) used as the aggregate; the sand fulfils the requirements of European norm EN 196-1 [21].

In Table 1 is given the compositions of the studied binding agents; the wastewater was added to these mixtures in the same state as it was taken from the treatment plant (liquid).

The fineness of the raw materials, i.e., Blaine specific surface area (Ssp), was assessed according to the method described in SR EN 196-6/2010 [22].

The chemical and mineralogical compositions of the raw materials were evaluated by the following:

(i) X-ray diffraction (XRD) analyses were performed in the range of 2θ = 5–65 degrees, using two diffractometers:

- Shimadzu XRD 6000 (Shimadzu, Kyoto, Japan), (λ = 1.5406 Å).
- Cubix (Malvern PANalytical, Almelo, The Netherlands) (λ = 1.5418 Å, with Rietveld refinement for assessment of the mineralogical composition).

(ii) X-ray fluorescence spectrometry was used to assess the oxide composition by means of an Axios equipment (Malvern PANalytical, Almelo, The Netherlands).

X-ray fluorescence spectrometry (Epsilon 5 equipment—Malvern PANalytical, Almelo, The Netherlands) was used to assess the heavy elements content in the raw materials used to obtain the binding agents.

The microstructure of materials was assessed by scanning electron microscopy (SEM) using a Quanta Inspect F type scanning electron microscope (Thermo Fisher—formerly FEI, Eindhoven, The Netherlands) with 1.2 nm resolution.

Complex thermal analyses (TG and DTG and DTA) were performed with a Shimatzu DTG-TA-60 (Shimadzu, Kyoto, Japan) with dynamic regime, in the temperature range 20–1000 °C, heating rate 10 °C/min., in oxidizing atmosphere.

The mechanical strengths were determined on pastes and mortars (prepared according to the method presented in SR EN 196-1/2016 [21]) cured for 7, 28 and 60 days, in the molds the first 24 h, then demolded and cured at 20 ± 1 °C in humid air (R.H. 90%). For the preparation of cementitious pastes, wastewater from the treatment of landfill leachate was used as the liquid component (Table 1). For mortars the binder/aggregate ratio was 1/3, and the water to binder ratio was 1.16 for A1, 1.09 for A2 and A3 and 0.5 for A4 and A5.

Mechanical strength tests were performed on a Matest machine (Matest, Treviolo, Italy). The compressive strengths were assessed on hardened prismatic specimens (40 mm × 40 mm × 160 mm) for the calculation of average mechanical strength (a minimum six compressive strength values were considered).

The hydration and hardening processes of the studied binding agents were assessed by X-ray diffraction (XRD), complex thermal analyses and scanning electron microscopy (SEM) on pastes hardened for 28 days.

The amount of heavy metals leached from the cementitious matrices with waste contents was determined according to the method presented in SR EN 12457-2 norm [23]. The leachates were obtained from immersing in double distilled water for 24 h the fine powders (<90 µm) resulting from the grinding of pastes hardened for 28 days. The leachates were analyzed by inductive coupled plasma optical emission spectrometry (ICP-OES); the analyses were performed on an inductively coupled plasma optical emission spectrometer, iCAP 6000 series, Thermo Scientific, USA.

## 3. Results and Discussion

In Table 2 is given the oxide composition (assessed by XRF) and mineralogical compositions (assessed by XRD with Rietveld refinement) of the INERCEM cements. As can be seen, the amounts of C_3_S, C_2_S and C_3_A were higher in INERCEM A, and the amounts of gypsum and calcite were higher in INERCEM E.

The specific Blaine surface areas of the materials used were 4063 cm^2^/g for INERCEM A, 4420 cm^2^/g for INERCEM E, 7538 cm^2^/g for fly ash and 1217 cm^2^/g for bottom ash. As expected, the bottom ash was much coarser compared with the cements and fly ash [12].

The results of X-ray fluorescence analyses (Table 3) showed that both types of ash used in this study were contaminated with heavy metals; in some cases the concentration of heavy metals was above the limit allowed for their storage in municipal waste landfills, according to Romanian and European legislation [24].

The wastewater from the treatment by reverse osmosis of the landfill leachate had a high content of some heavy metals (Table 3); therefore, it could not be released into natural receptors [25].

The oxide compositions of the coal ash used in this study are presented in Table 4. The fly ash was determined to be a type F with high silica and alumina content, and the bottom ash was type C with a higher calcium content. The higher loss on ignition assessed for coal bottom ash also suggested a higher coal content compared to fly ash.

The crystallinity degree, mineralogical composition and morphological characteristics of the materials used for the binding agents were assessed by X-ray diffraction (XRD) and scanning electron microscopy (SEM) analysis.

**Figure 1:**
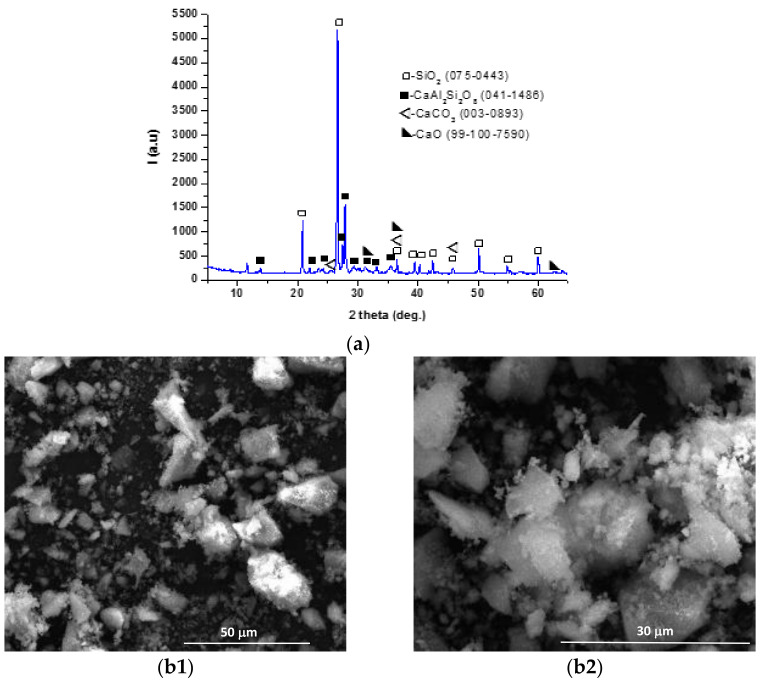
XRD pattern (**a**) and SEM images (**b1**—×2k, **b2**—×5k) for coal bottom ash.

As can be seen from Figure 1a, the coal bottom ash contains quartz and anorthite as main crystalline mineralogical phases and lime and calcite in small quantities; the SEM images (Figure 1b1,b2) show the presence of particles with polyhedral shapes, with average sizes below 50 µm.

**Figure 2:**
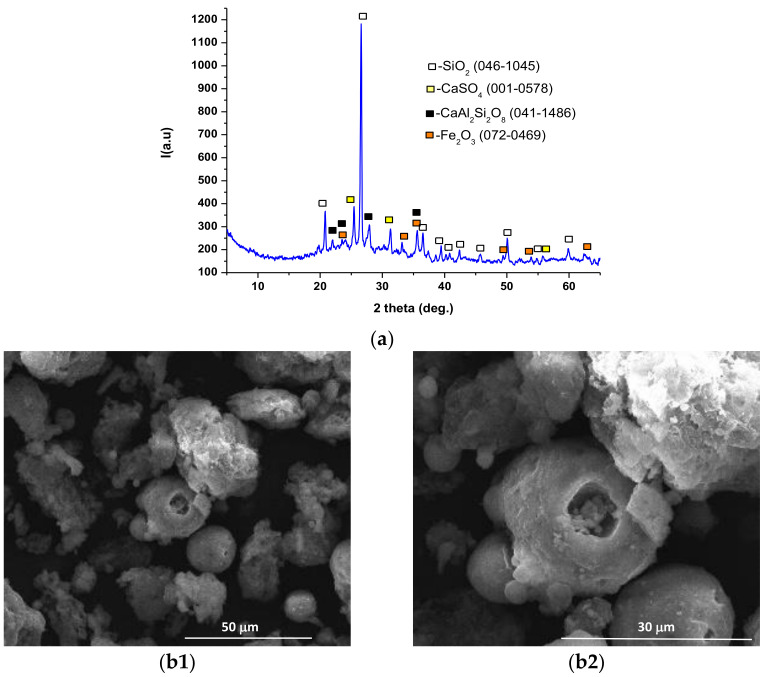
XRD pattern (**a**) and SEM images (**b1**—×2k, **b2**—×5k) for fly ash.

Fly ash is a material with a lower crystallinity compared to coal bottom ash; the presence of a phase with lower crystallinity was suggested by the halo present between 2θ = 20–40 degrees on the XRD pattern (Figure 2a). The main crystalline mineralogical phases assessed in the fly ash were quartz, anorthite, anhydrite and hematite (Figure 2a). The SEM images presented in Figure 2(b1,b2), show the presence of cenospheres (round hollow particles) and irregularly shaped particles, with average sizes below 50 µm.

**Figure 3:**
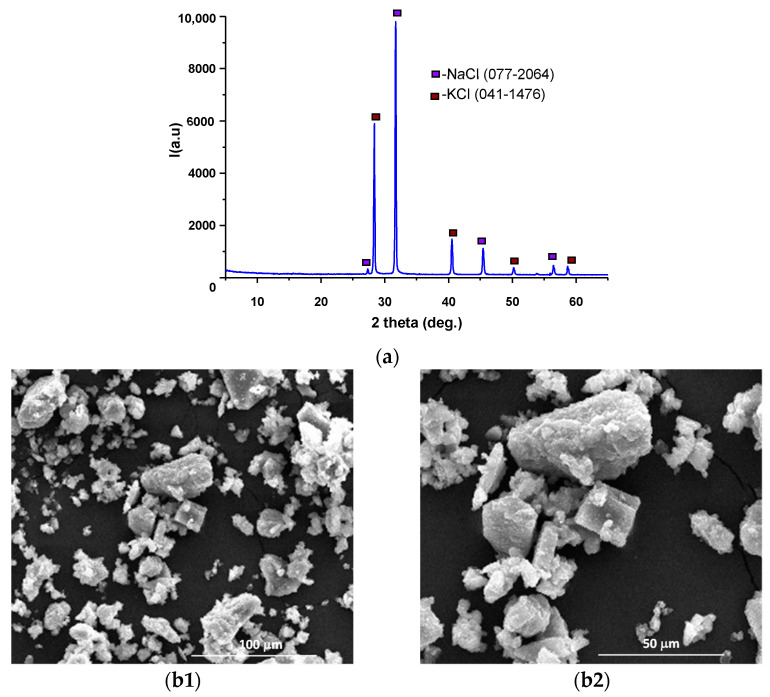
XRD pattern (**a**) and SEM images (**b1**—×1k, **b2**—×2k) for the solid residue resulting from the evaporation of wastewater.

The XRD pattern of wastewater (previously kept at 40 °C up to constant mass) showed the presence of two crystalline compounds, namely sodium and potassium chlorides (Figure 3a); the typical structures of these salts are assessed in the SEM images presented in Figure 3(b1,b2) [26,27,28,29,30].

**Figure 4:**
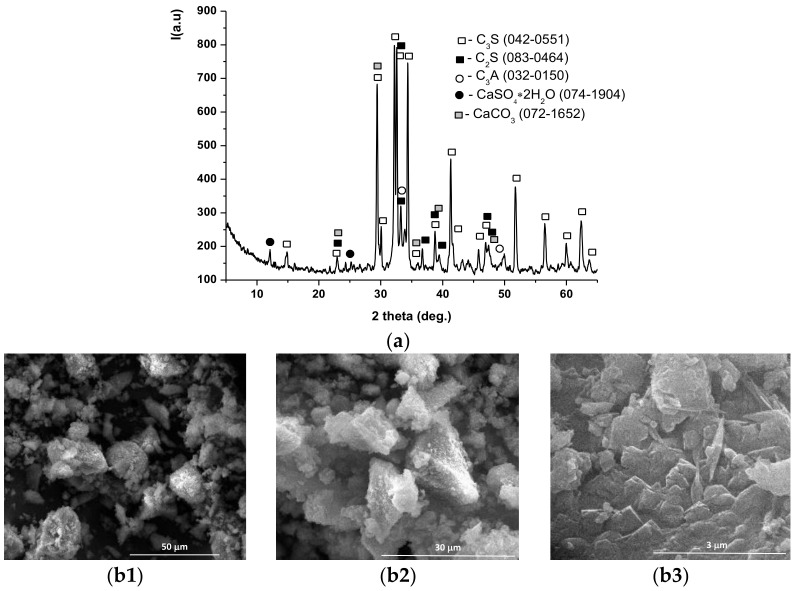
XRD pattern (**a**) and SEM images (**b1**—×2k, **b2—**×5k, **b3—**×50k) for INERCEM A.

**Figure 5:**
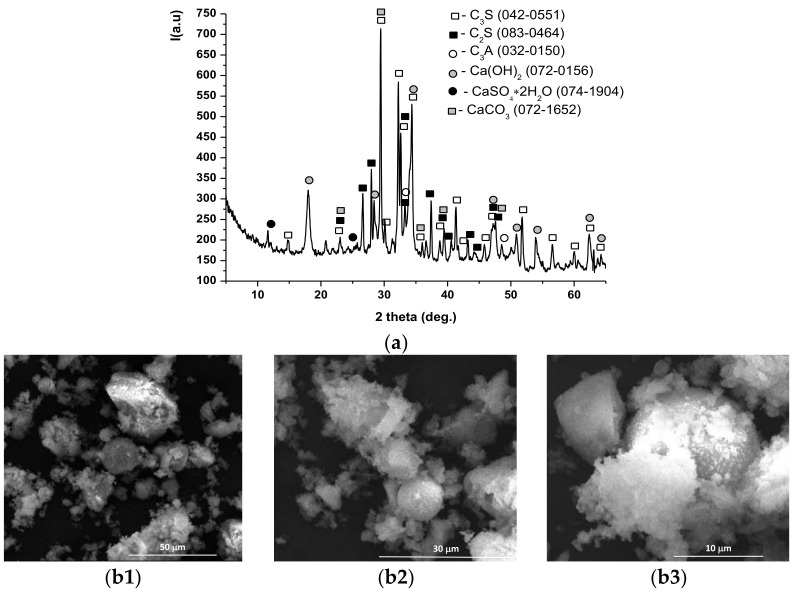
XRD pattern (**a**) and SEM images (**b1**—×2k, **b2**—×5k, **b3**—×10k) for INERCEM E.

The main crystalline mineralogical compounds assessed in INERCEM A and E were calcium silicates (alite-C_3_S, belite-C_2_S), tricalcium aluminate (C_3_A), gypsum (CaSO_4_·2H_2_O) and calcite (CaCO_3_)— Figure 4a and Figure 5a; in addition, INERCEM E also contained a significant amount of calcium hydroxide-Ca(OH)_2_—Figure 5a.

Scanning electron microscopy images showed the presence of the following mineralogical phases identified by XRD: C_2_S (crystals with quasi-spherical morphology), C_3_S (crystals with polyhedral shapes) and C_4_AF as an interstitial phase (Figure 4(b1–b3) and Figure 5(b1–b3)).

**Figure 6:**
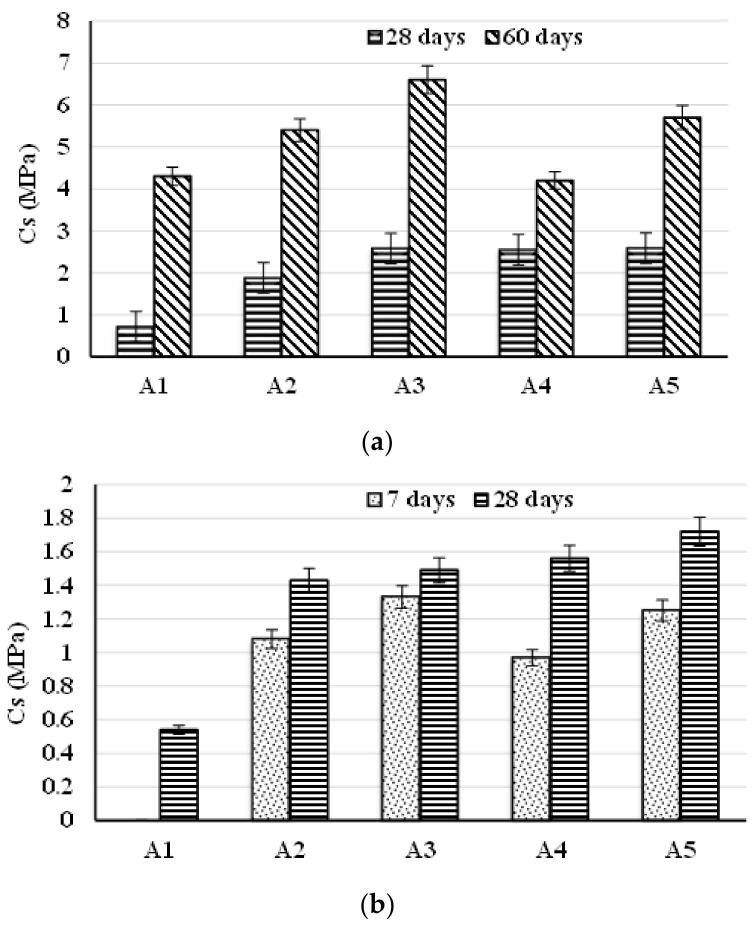
Compressive strength of pastes (**a**) and mortars (**b**).

The values of compressive strengths are presented in Figure 6a,b. As can be seen, although the values of compressive strength were small (around 0.7–2.7 MPa after 28 days), they were higher compared with the minimum strength set by USEPA (i.e., 0.35 MPa) [31,32,33,34,35], proving that the liquid waste was chemically bonded not only absorbed. Moreover, the compressive strength values increased with the increase of the hardening period.

**Figure 7:**
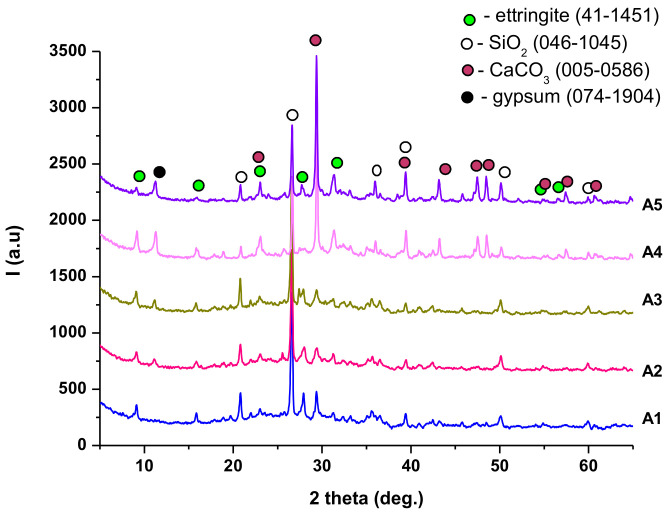
XRD patterns of cement pastes A1–A5 hardened for 28 days.

**Figure 8:**
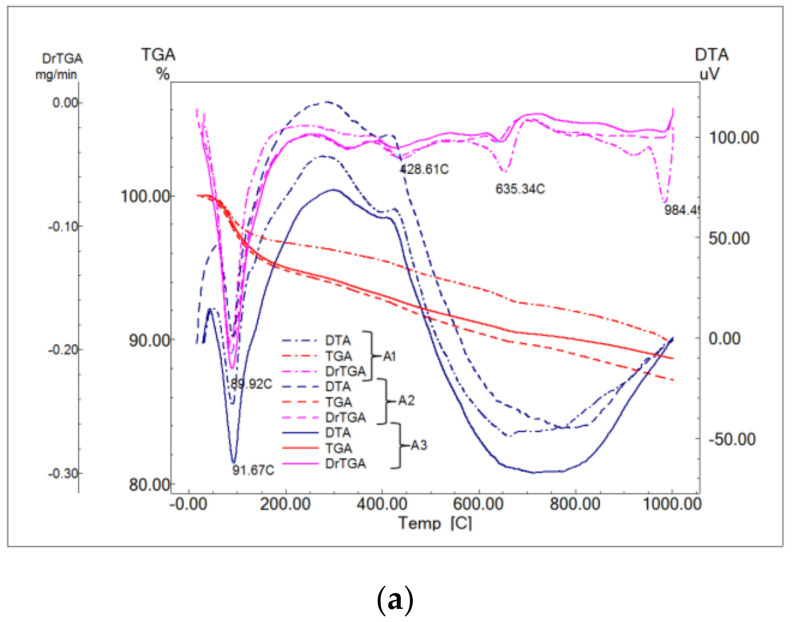

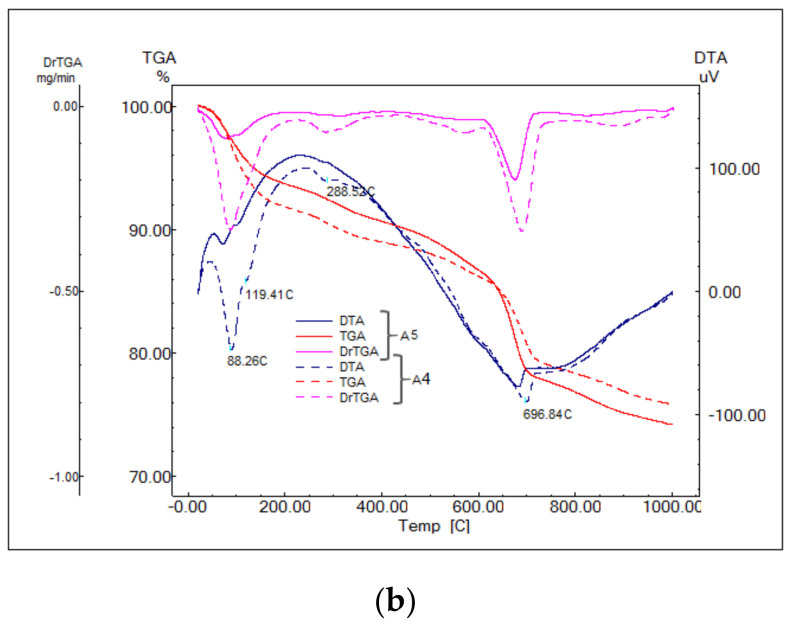
Thermal analyses of A1–A3 binders (**a**) and A4–A5 binders (**b**) hardened 28 days.

XRD patterns of binding pastes A1–A5 hardened for 28 days, presented in Figure 7, highlighted the presence of quartz (compound assessed in both ashes) and gypsum (present in INERCEM); one could also assess the formation of ettringite (resulting from the hydration of INERCEM) and CaCO_3_, which was most likely formed by the carbonation of Ca(OH)_2_ with atmospheric CO_2_. These results were also supported by data obtained by complex thermal analysis (DTA-TG)—Figure 8.

DTA curves (Figure 8) showed the presence of endothermic effects, accompanied by mass loss, attributed to the following processes [36]:

- Endo-effect between 88 and 92 °C determined by the dehydration of ettringite with different degrees of crystallinity;
- Effects at approx. 119, 188 and 288 °C due to the decomposition of calcium silicate hydrates with a low degree of crystallinity;
- Decomposition of calcium hydroxide effects at approx. 428 and 555 °C;
- Effects at approx. 635 and 697 °C from decomposition of calcium carbonate with a low degree of crystallinity;
- Effect at approx. 984 °C of decomposition of calcium carbonate with a higher degree of crystallinity.

**Figure 9:**
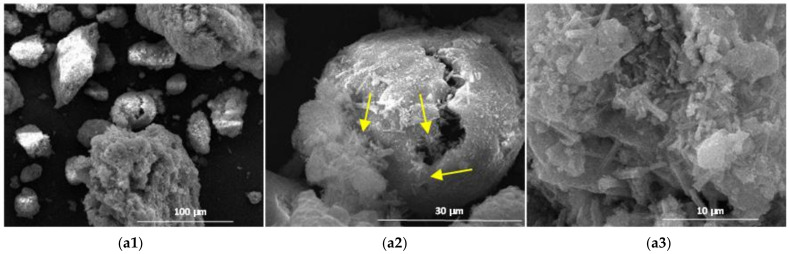
SEM images of the 28-days hardened A1 binder paste (**a1—**×1k, **a2—**×5k, **a3—**×10k).

**Figure 10:**
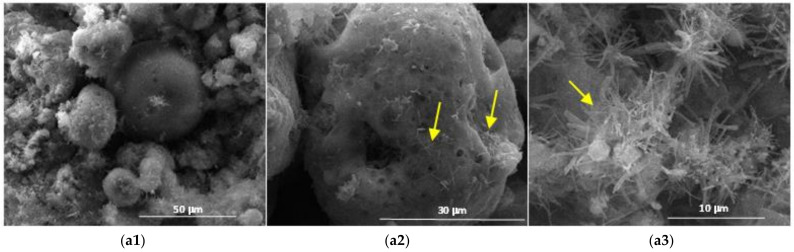
SEM images of the 28-days hardened A2 binder paste (**a1**—×2k, **a2**—×5k, **a3**—×10k).

**Figure 11:**
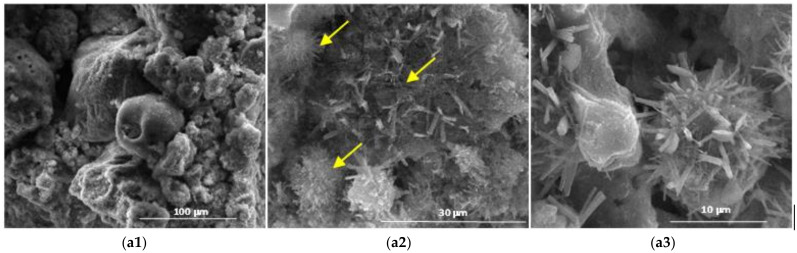
SEM images of the 28-days hardened A3 binder paste (**a1**—×1k, **a2**—×5k, **a3**—×10k).

**Figure 12:**
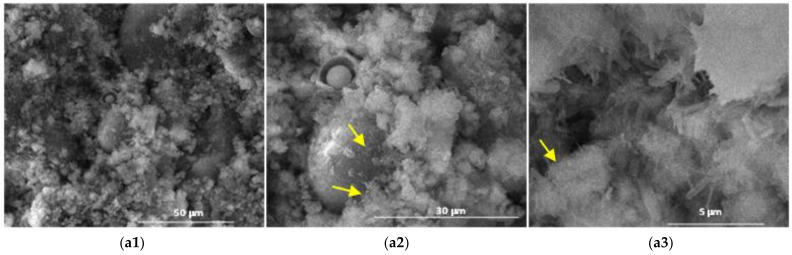
SEM images of the 28-days hardened A4 binder paste (**a1**—×2k, **a2**—×5k, **a3**—×40k).

**Figure 13:**
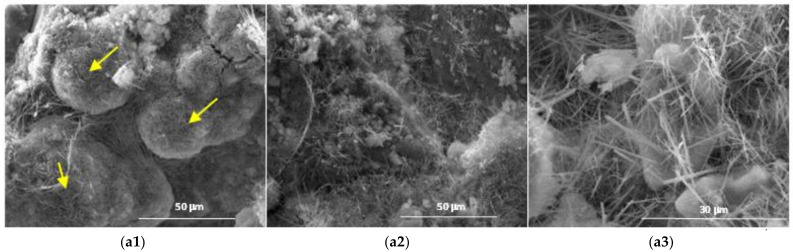
SEM images of the 28-days hardened A5 binder paste (**a1**,**a2**—×2k, **a3**—×5k).

The results obtained by scanning electron microscopy (Figure 9, Figure 10, Figure 11, Figure 12 and Figure 13) were in good correlation with the results obtained by XRD and DTA-TG; these images highlighted the presence of prismatic and acicular ettringite crystals, carbonate phases with undefined morphology and calcium silicate hydrates with acicular morphology. At the surface of the spherical ash particles (see arrows in Figure 9, Figure 10, Figure 11, Figure 12 and Figure 13), one could observe the presence of hydrates (most probably calcium silicate hydrates), which could result also in the pozzolanic reaction, confirming the higher reactivity of this vitreous phase.

The results of the levigation test (Table 5) showed good immobilization of heavy metals in the studied cementitious matrices; all leachates had heavy metal concentrations well below the maximum limits stipulated in specific legislation—Governmental Order 95/2005 [24]. This important reduction of heavy metal leaching when encapsulated in the studied cementitious matrices can be explained by the high capacity of ettringite to integrate heavy metals into its structure. In addition, the gel-like calcium silicate hydrates can adsorb heavy metals on their surface [7,9,37,38,39,40].

## 4. Conclusions

Fly and bottom ash generated by coal combustion in an electric power plant with a high content of heavy metals were used in mixtures with specially designed cements for waste/contaminated soil treatment (INERCEM) to solidify/stabilize wastewater with high concentrations of heavy metals (resulting from the treatment of a landfill leachate formed in a municipal waste landfill).

All cementitious systems were efficient for the solidification/stabilization of this liquid waste and could reduce the leaching potential of heavy metals present in wastewater and coal ash. Therefore, these ashes can be valorized for this type of application and cease to be a source of environmental pollution.

X-ray diffraction (XRD) and thermal complex analysis (DTA-TG) were used to assess the nature of compounds formed in these cementitious systems during the hydration and hardening processes; ettringite, calcium silicate hydrates and CaCO_3_ were the main compounds assessed by these methods. The microstructure of hardened specimens was assessed by scanning electronic microscopy (SEM); the presence of hydrate phases, at the surface of cenospheres present in fly ash, proved the higher pozzolanic reactivity of this phase.

All studied materials (pastes or mortar) developed mechanical strengths of approx. 0.7–2.7 MPa after 28 days of hardening; for pastes, these values increased for longer curing times (60 days) up to 6.6 MPa, proving that the heavy metals from liquid and solid wastes are chemically bound in the studied cementitious matrices. Based on the compression strengths assessed in this study, the potential utilization of these materials could be as an inert material to close waste landfills or as base layers for roads.

## Figures and Tables

**Table 1:** Composition of studied cementitious systems.

Symbol Cementitious Systems	Fly Ash	Bottom Ash	INERCEM E	INERCEM A	Wastewater *
	wt.%				
**A1**	51	0	4	0	45
**A2**	47	0	6	2	45
**A3**	47	0	4	4	45
**A4**	0	52	6	2	40
**A5**	0	47	4	4	45

* the water content is 97.4%.

**Table 2:** Oxide and mineralogical composition of INERCEMs (A and E).

INERCEM	Oxide Composition (wt.%)							
	SiO_2_	Al_2_O_3_	Fe_2_O_3_	CaO	MgO	Na_2_O	K_2_O	SO_3_
**A**	19.85	6.5	2.75	64.38	1.4	0.54	1.07	3.49
**E**	24.58	8.75	4.35	52.03	1.78	0.53	1.25	2.19
**INERCEM**	**Mineralogical Composition (wt.%)**							
	**C_3_S**	**C_2_S**	**Tricalcium aluminate**			**C_4_AF**	**Gypsum**	**Calcite**
			cC_3_A	oC_3_A	C_3_A			
**A**	67.25	8.14	4.25	3.46	8.97	1.47	0	2.97
**E**	33.8	5.96	3.05	1.53	4.58	5.08	1.16	7.54

C—CaO, S—SiO_2_, A—Al_2_O_3_, F—Fe_2_O_3_.

**Table 3:** The concentrations of heavy metals in wastewater and coal ash.

Material/Element	H_2_O	Cl	As	Cr	Cd	Ni	Cu	Zn	Ba	Hg	Mo	Pb	Sb	Se	V	Mn
	wt.%		mg/kg													
Fly ash	0.5	0	27	73	0	67	55	77	274	0	0	22	0	0	199	305
Bottom ash	5.0	0.1	0	126	0	105	35	1151	821	1	15	27	23	0	16	205
**Material/Element**	**H_2_O**	**Cl**	**As**	**Cr**	**Cd**	**Ni**	**Cu**	**Zn**	**Ba**	**Hg**	**Mo**	**Pb**	**Sb**	**Se**	**V**	**Mn**
	**wt.%**		**mg/dm^3^**													
Max. conc. allowed by specific legislation [25]	**-**	**0.5**	**0.1**	**1**	**0.2**	**0.5**	**0.1**	**0.5**	**-**	**0.05**	**0.1**	**0.2**	**-**	**0.1**	**-**	**1**
Wastewater	97.4	0.3	0.0	4.1	0	2.3	2.7	4.7	12	0	0	0	0	0	2.1	4.2

**Table 4:** Oxide composition of coal ash.

Material	Oxide composition (wt.%)										
	L.O.I *	SiO_2_	Al_2_O_3_	Fe_2_O_3_	CaO	MgO	SO_3_	K_2_O	Na_2_O	P_2_O_5_	ZnO
Bottom ash	8.8	34.6	3.7	10.0	19.1	1.6	7.3	1.1	1.7	2.2	1.3
Fly ash	3.4	52.9	22.1	8.5	7.4	2.5	0.5	1.5	0.7	0.18	0.02

* L.O.I.—Loss on ignition.

**Table 5:** Concentration of heavy elements assessed in leachates.

Element	As	Ba	Cd	Cr	Hg	Mo	Sb	Se	Zn	Cu	Ni	Pb
Maxim. allowed (mg/kg) [24]	25	300	5	70	2	30	5	7	200	100	40	50
**A1**	0	5.4	0	1.15	0.2	0.25	1.35	1.2	0	2.1	0	0.2
**A2**	0	5.5	0.1	0.95	0.15	0.1	1.7	1.2	0.5	2.1	0	0.1
**A3**	0	5.9	0.15	1.3	0.2	0.2	1.6	1.2	1.25	2.1	0	0.2
**A4**	0	5.85	0	1.55	0.2	0.2	1.65	1.3	0.5	1.35	0	0.3
**A5**	0	6.25	0.2	1.5	0.2	0.25	1.45	1.3	0.65	1.3	0	0.45

## Data Availability

Not applicable.
